# Revealing the Structure of Sheer N-Acetylglucosamine, an Essential Chemical Scaffold in Glycobiology

**DOI:** 10.1021/acs.jpclett.4c02128

**Published:** 2024-10-07

**Authors:** Elena R. Alonso, Aran Insausti, Isabel Peña, Miguel Sanz-Novo, Raúl Aguado, Iker León, José L. Alonso

**Affiliations:** † Grupo de Espectroscopia Molecular (GEM), Edificio Quifima, Área de Química-Física, Laboratorios de Espectroscopia y Bioespectroscopia, Parque Científico UVa, Unidad Asociada CSIC, Universidad de Valladolid, 47011 Valladolid, Spain; # Departamento de Química Física, Facultad de Ciencia y Tecnología, Universidad del País Vasco, Barrio Sarriena s/n, 48940 Leioa, Spain; ◊ Departamento de Química Física y Química Inorgánica, Facultad de Ciencias, Universidad de Valladolid, 47011 Valladolid, Spain

## Abstract

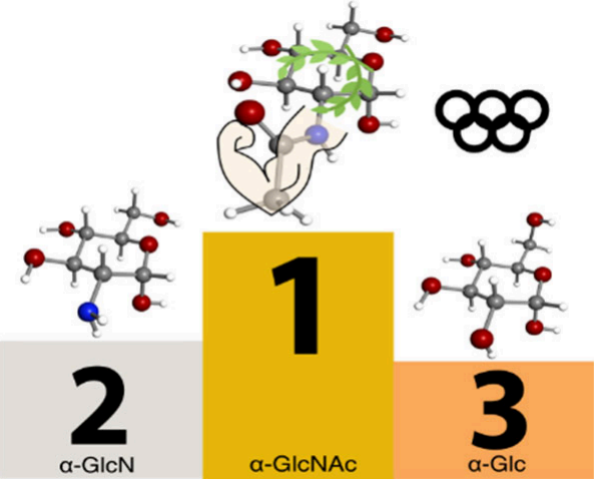

We explored the conformational landscape of N-acetyl-α-d-glucosamine (α-GlcNAc), a fundamental chemical scaffold in glycobiology. Solid samples were vaporized by laser ablation, expanded in a supersonic jet, and characterized by broadband chirped pulse Fourier transform microwave spectroscopy. In the isolation conditions of the jet, three different structures of GlcNAc have been discovered. These are conclusively identified by comparing the experimental values of the rotational constants with those predicted by theoretical calculations. The conformational preferences are controlled by intramolecular hydrogen bond networks formed between the polar groups in the acetamido group and the hydroxyl groups and dominated in all cases by a strong OH···O=C interaction. We reported an exception to the gauche effect due to the enhanced stability observed for the Tg^+^ conformer. All the structures present the same disposition of the acetamido group, which explains the highly selective binding of N-acetylglucosamine with different amino acid residues. Thus, the comprehensive structural data provided here shall help to shed some light on the biological role of this relevant amino sugar.

The amino sugar N-acetyl-d-glucosamine (C_8_H_15_NO_6_; GlcNAc, see Figure 1) plays an essential biological role in the surface of various cell types, ranging from bacteria to humans.^1,2^ GlcNAc acts as the monomer unit in fungal cell surfaces made up of the polysaccharide chitin,^3,4^ and it takes part in various oligosaccharides in the extracellular matrix of animal cells.^5^ There is increasing evidence that GlcNAc affects cell signaling^6^ and has an impact on the virulence properties of microbes and host cells.^7−9^ It influences protein glycosylation by regulating cell signaling pathways and participating in O-GlcNAcylation with Ser and Thr amino acid residues of cytosolic and nuclear proteins.^10^ GlcNAc is also being used as a docking target for new biomimetic receptors due to its presence in N-glycans exposed on the surface of enveloped viruses, such as coronaviruses.^11^ Additionally, it has antitumor and immunoregulatory effects.^12^ All the plethora of cellular processes in which GlcNAc is involved is related to its intrinsic or primitive structure and all the possible interactions it can establish with its surroundings, which are determined by the topology of the molecule.

**Figure 1:**
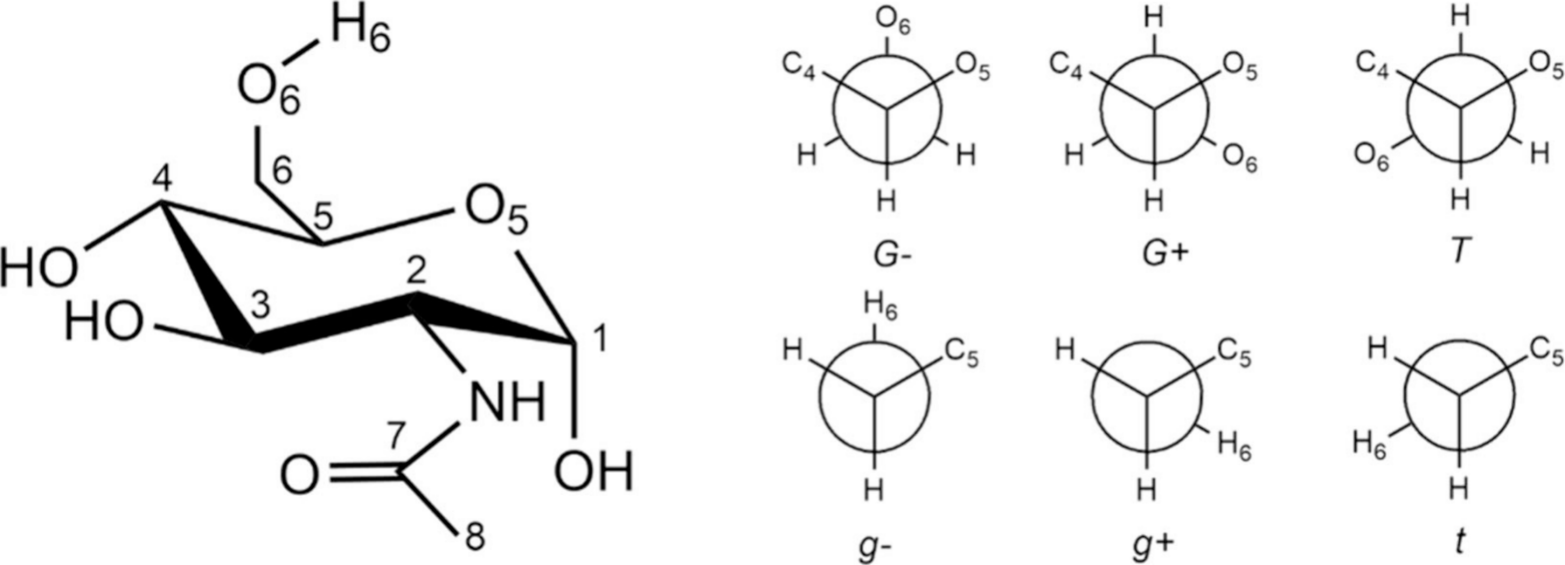
^4^C_1_ chair configuration of α-N-acetyl-d-glucosamine (α-GlcNAc) and the Newman projections of plausible conformations of the hydroxymethyl group around the C_5_–C_6_ (G, G^+^, T) and C_6_–O_6_ (g^–^, g^+^, t) bonds.

Despite its remarkable importance, only a few studies have been conducted on the structure of GlcNAc. The crystal structure was studied by X-ray diffraction^13,14^ and FT-IR spectroscopy in an aqueous solution,^15^ revealing that the most abundant anomer is α. Molecular dynamics studies on the behavior of GlcNAc in condensed phases have also been performed, combining NMR experimental data with computational methods.^16,17^ The conformation of the N-acetyl side-chain of GlcNAc in solution has been efficiently predicted using explicit solvent molecules or specifically designed theoretical models.^16,18,19^ In the gas phase, only the GlcNAc derivate phenyl N-acetyl-β-d-glucosamine was studied by infrared ion depletion spectroscopy.^20^ Although all the above constitute valuable structural information on GlcNAc, no experimental data on the conformational behavior of its sheer form has been reported so far. It is well-known that structures adopted in condensed phases are guided by strong interactions of hydrogen bonds, both inter- and intramolecular, with the solvent or within the molecule. To understand the structure of GlcNAc in their native biological conditions, it is often imperative to unveil it without intermolecular interactions, avoiding alterations of their intrinsic conformational preferences.

Gas-phase high-resolution spectroscopic techniques, such as rotational spectroscopy, can resolve individual conformational signatures without interference from the media. These techniques can provide accurate structural information directly comparable to the in-vacuo theoretical predictions, leading to an unequivocal identification of the most abundant structural species. However, working with gas-phase amino sugars can be challenging due to vaporization difficulties related to the inherently labile nature of their solid samples. GlcNAc, for instance, is a thermolabile solid with a very high melting point (211 °C, decomposition), making it difficult to transfer to the gas phase using conventional heating methods. Additionally, GlcNAc may suffer from photofragmentation problems when submitted to a laser vaporization procedure.^21^ While the laser-ablated rotational studies of its intimately related α-d-glucosamine and α/β-d-glucose have already been performed,^22,23^ the high-resolution study of GlcNAc has remained an arduous endeavor for rotational spectroscopy. Our laboratory has made significant progress in enhancing the laser ablation microwave instrumentation^24,25^ in an attempt to minimize the effects of laser-induced fragmentation. It has enabled us to conduct the first-ever rotational spectroscopy analysis of α-GlcNAc. To examine the GlcNAc molecule effectively, we have employed a combination of laser ablation (LA) and chirped pulse Fourier transform microwave spectroscopy (CP-FTMW)^26,27^ which has been successfully used to analyze a wide range of biomolecular systems.^28^

Our study focuses on the α anomer, the crystal’s most abundant anomer. α-GlcNAc differs structurally from the parent α-d-glucose by replacing the hydroxyl group on C_2_ with an acetamido group. The pyranose ring can adopt either ^1^C_4_ or ^4^C_1_ chair configurations, with the latter being dominant. The hydroxymethyl −CH_2_OH and acetamido −NHC(=O)CH_3_ groups are equatorial in this ^4^C_1_ chair configuration, making it energetically favored. The flexibility of the hydroxymethyl group can lead to three staggered forms, G^–^, G^+^ (gauche), and T (trans), represented by the O_6_–C_6_–C_5_–O_5_ dihedral angle with values of approximately −60°, 60°, and 180°, respectively. Similarly, the symbols g^–^, g^+^, and t describe the conformations defined by the H_6_–O_6_–C_6_–C_5_ dihedral angle (see Figure 1). As part of our experiment, we conducted a conformational search using a fast molecular mechanics method (MMFFs)^29^ to explore the conformational landscape of α-GlcNAc. Two search algorithms were employed, a “Large scales Low Mode” algorithm and a Monte Carlo based search implemented in the Macromodel.^30^ Then, we geometrically optimized the structures obtained using various DFT, double-hybrid DFT, and ab initio methods.^31−33^ (See Tables S1–S3 and Figure S1 of the Supporting Information). The spectroscopic parameters predicted for the three lower energy conformers, labeled as G^+^g^–^, Tg^+^, and G^–^g^+^, are listed in Table 1.

**Table 1:** Experimental and Predicted Rotational Parameters of α-GlcNAc

	Experimental			Theory^a^		
Parameter^b^	Rotamer I	Rotamer II	Rotamer III	G^+^g^–^	Tg^+^	G^–^g^+^
*A*/MHz	1145.36429(68)^c^	1205.1720(13)	1085.95451(56)	1140.7	1201.4	1078.5
*B*/MHz	357.67105(33)	353.76606(30)	368.17308(20)	357.6	353.5	368.0
*C*/MHz	296.33860(31)	296.30157(15)	318.66494(13)	295.6	295.2	318.6
Δ_J_ /Hz	2.91(66)	...	...			
*|μ*_*a*_*|/|μ*_*b*_*|/|μ*_*c*_*|* D	Y/Y/N^d^	Y/Y/N	N/Y/N	2.8/4.4/0.3	3.6/4.0/0.4	2.4/5.5/1.6
N	75	41	44	...	...	...
σ_rms_ /kHz	16.8	14.4	13.8	...	...	...
Δ*E*_Tot_/ cm^–1^	...	...	...	79	0.0	121
Δ*G*/cm^–1^	...	...	...	0.0	80	62

a Calculated at the B2PLYP-D3BJ/6-311++G(d,p) level of the theory.

b *A*, *B*, and *C* are the rotational constants; Δ_*J*_ is one of the quartic centrifugal distortion constants (*A*-reduction); |μ_*a*_|, |μ_*b*_|, and |μ_*c*_| are the absolute values of the electric dipole moment components along the inertial axis *a*, *b*, *c*; *N* represents the number of distinct frequency lines in the fit; σ_rms_ is the root-mean-square deviation of the fit; *ΔE*_Tot_ and *ΔG* represent the total relative energy (E+E_ZPE_) and Gibbs free energy (*T* = 298 K), respectively, relative to the global minimum.

c The numbers in parentheses are 1σ uncertainties in the last decimal digit units.

d Experimental observation or nonobservation of a given type of rotational transition.

We noticed significant photofragmentation effects after analyzing the congested LA-CP-FTMW broadband rotational spectrum shown in Figure S2. To minimize the photofragmentation, we interactively adjusted the experimental conditions, such as laser fluence, stagnation pressure, and time delays between the gas, laser, and microwave pulses, until the intensity of the known photofragment lines was minimized. We then eliminated all undesired lines, leading us to the still very overcrowded spectrum in Figure 2a. This is the target spectrum of our research where we can identify α-GlcNAc signals although the spectra of other unknown photofragments prevail.

**Figure 2:**
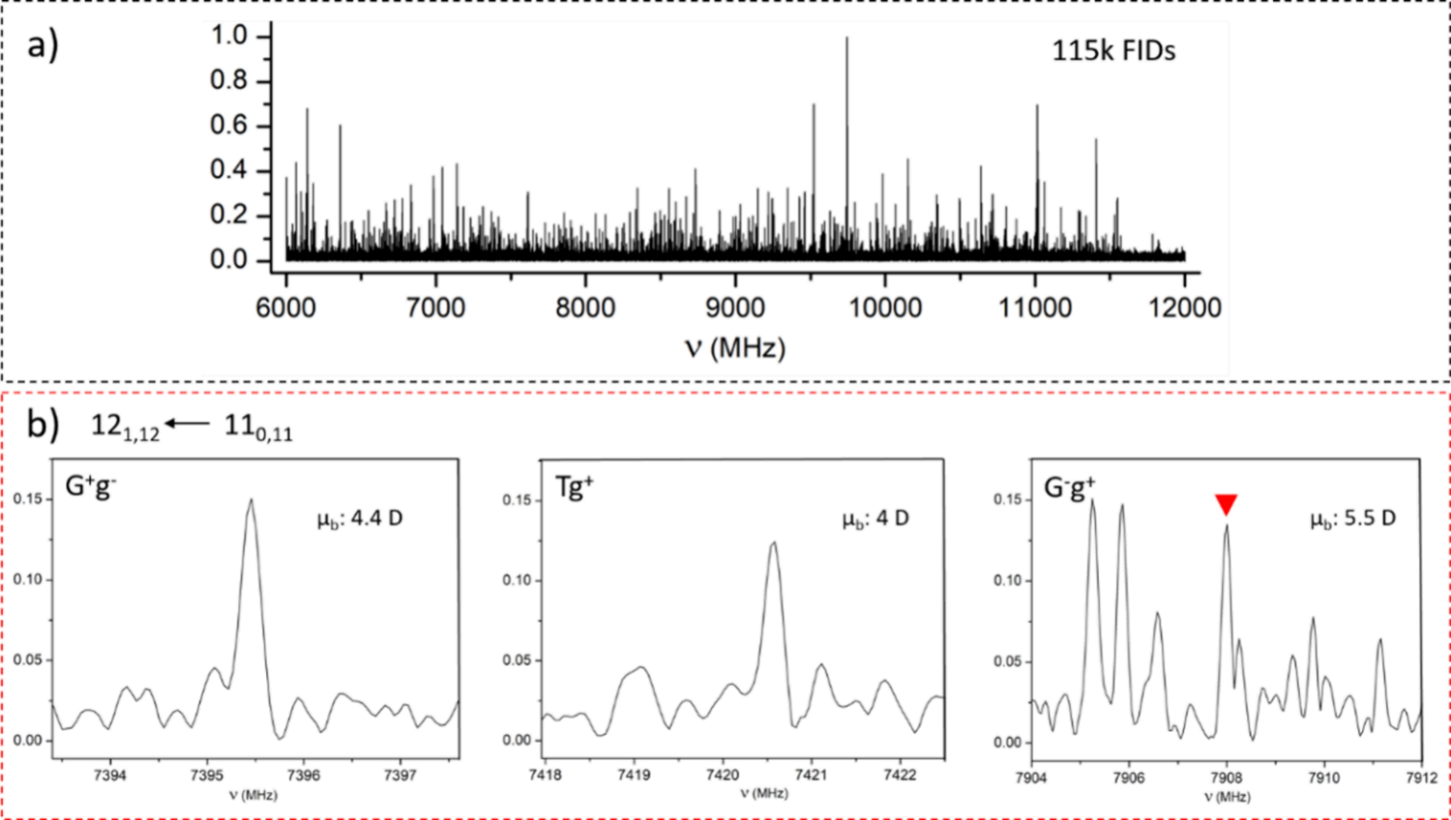
(a) Experimental LA-CP-FTMW rotational spectrum of α-GlcNAc in the 6–12 GHz frequency range, already cleaned from photofragmentation lines. (b) μ_*b*_-R-Branch transition 12_1,12_ ← 11_0,11_ displayed for the three identified conformers.

Guided by the theoretical rotational parameters in Table 1, we disentangled the rotational spectrum of α-GlcNAc from those of other unknown species. We identified 75 rotational transitions corresponding to μ_*a*_- and μ_*b*_-type *R*-branch progressions (see Table S4 of the Supporting Information) attributed to a first rotamer I. They were fitted to a Watson’s A-reduced semirigid rotor Hamiltonian,^34^ which led to the set of rotational constants listed in the first column of Table 1. These values nicely match the ones predicted for G^+^g^–^ conformer at the double hybrid B2PLYP-D3BJ/6-311++G(d,p) level of theory, also collected in Table 1. We repeated the search-measure-fit procedure to identify rotamers **II** (41 lines, Table S6 of SI) and **III** (44 lines, Table S5 of SI) in the spectrum, whose experimental rotational constants, also listed in Table 1, correspond to those predicted for Tg^+^ and G^–^g^+^ conformations, respectively. Scale factors ranging from 0.993 to 0.999 brought the predicted rotational constants to coincide with the experimental ones, further supporting the global assignment and reflecting the excellent match between theory and experiment. Hence, the B2PLYP structures presented in Figure 3 accurately represent the structures for the three identified conformers. Their Cartesian coordinates are reported in Tables S7–S9 of the SI. It is worth noting that the hyperfine nuclear quadrupole structure caused by the ^14^N nucleus has not been detected in the measured transitions. This is due to the high J values of the measured transitions.

**Figure 3:**
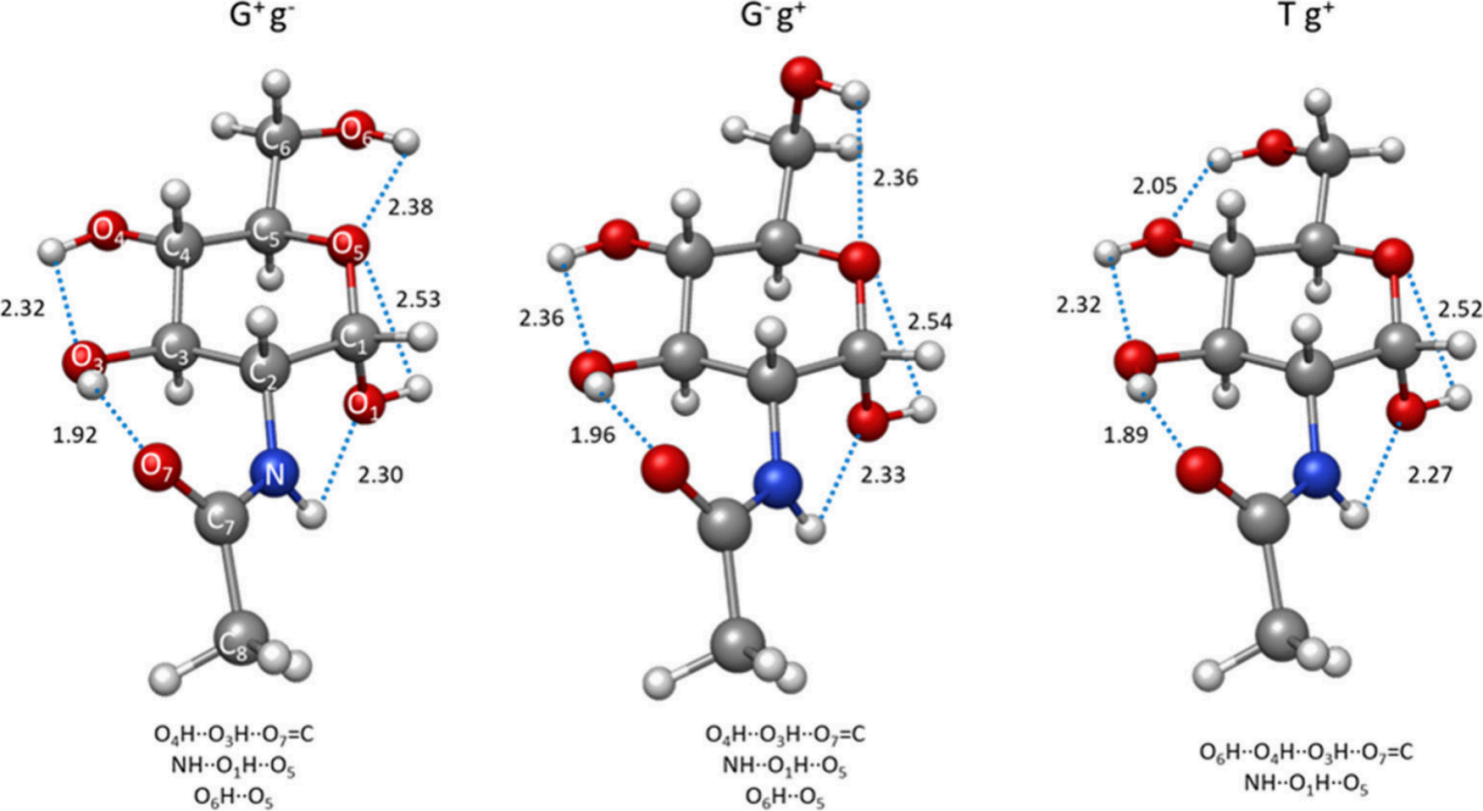
Three observed conformers of α-GlcNAc showing the intramolecular hydrogen bonding distances (B2PLYP-D3BJ/6-311++G(d,p) level of the theory) given in Å.

α-GlcNAc adopts three different conformations, all with a ^4^C_1_ ring configuration and the anomeric OH group directed toward the axial position due to the anomeric effect.^35,36^ The other OH and – NHC(=O)CH_3_ groups are all located at equatorial positions, forming hydrogen bond chains reinforced by sigma-hydrogen bond cooperativity.^37^ This phenomenon is associated with hydrogen bonding networks between groups that act simultaneously as proton donors and acceptors. The G^+^g^–^ and G^–^g^+^ conformers display a cooperative hydrogen bond pattern, extending in a counterclockwise direction, specifically O_4_H··O_3_H··O_7_=C and NH··O_1_H··O_5_. Separately, their hydroxymethyl groups are involved in another, noncooperative O_6_H··O_5_ interaction. The Tg^+^ conformer exhibits a similar counterclockwise cooperative hydrogen bonding pattern. However, in this case, the hydroxymethyl group is also a part of the cooperative hydrogen bond sequence O_6_H··O_4_H··O_3_H··O_7_=C_._ Thus, the enhanced stability of the observed conformations is primarily attributed to the cooperative network of hydrogen bonds, particularly to the strong O_3_H···O=C_7_ interaction taking place only in the observed counterclockwise arrangement. The conformational diversity of α-GlcNAc must be only considered in terms of the spatial disposition of its flexible CH_2_OH side chain to form the three staggered G^+^, G^–^ and T configurations associated with the C_6_–O_6_–C_5_–O_5_ torsional angle.

The discovery of T configurations in α-GlcNAc is truly remarkable. Numerous experimental studies on glucopyranosides in condensed phases have shown that G^+^ and G^–^ conformers have approximately equal populations, with hardly any T species.^38^ This tendency of glucopyranosides to adopt gauche conformations is known as the gauche effect,^39^ generally accepted as a solvent-dependent phenomenon.^40^ However, our gas-phase experiment involving α-d-glucose (α-Glc), α-d-glucosamine (α-GlcN),^22,23^ and now α-GlcNAc revealed the existence of T configurations. One of the challenges in discovering these forms in condensed phases arises from the intermolecular interactions that usually mask the intrinsic properties of glucopyranosides, including their ability to stabilize the Tg^+^ conformer. The intrinsic conformational choices of α-GlcNAc can only be unveiled in the isolated gas phase conditions. It highlights the importance of exploring different experimental methods and environments in scientific research.

The relative abundance of the three G^+^g^–^, G^–^g^+^, and Tg^+^ conformers was determined by analyzing the line intensities of selected transitions in conjunction with predicted dipole moment components (see Figure 2b).^41^ The experimental results and the predicted theoretical population ratios are presented in Figure 4, which shows that considering the energetic parameters, the B3LYP-D3BJ predictions better match the experimental abundances.

**Figure 4:**
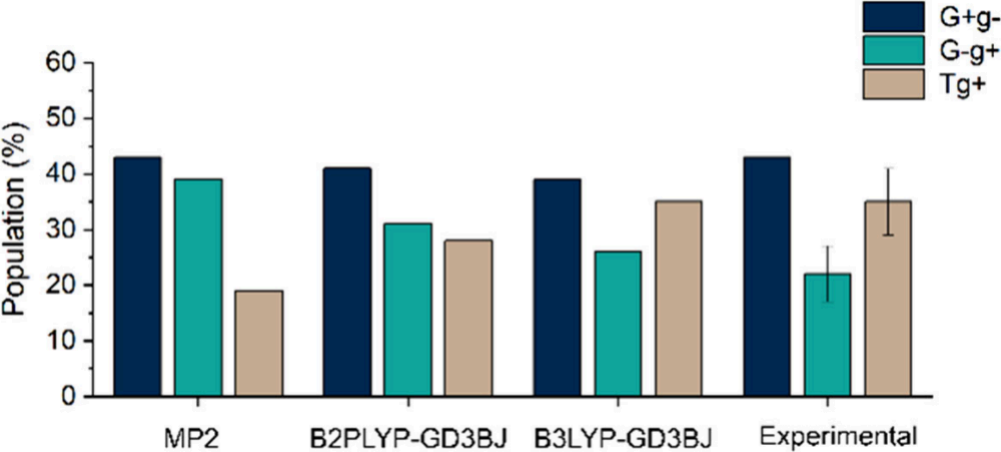
Theoretical and experimental relative abundance of α-GlcNAc conformers. MP2 predictions are not in agreement with the derived experimental population ratios, underestimating the relative stability of Tg^+^ and overestimating the stability of G^–^g^+^ (G^+^g^–^:G^–^g^+^:Tg^+^ ≈ 43:39:19). Curiously, only the B3LYP-D3BJ match completely with our experimental results of α-GlcNAc (G^+^g^–^:G^–^g^+^:Tg^+^ ≈ 39:26:35). The B2PLYP-D3BJ values (G^+^g^–^:G^–^g^+^:Tg^+^ ≈ 41:31:28) are in between those of MP2 and B3LYP. All calculations were done using a 6-311++G(d,p) basis set. The error bars represent the standard deviation of the values obtained at different transitions, common to the three conformers. Note that the most populated conformer is used as a reference, so it does not have an associated error.

Figure 5 shows the experimental relative abundance of counterclockwise conformers of α-GlcNAc compared to those previously reported for α-Glc and α-GlcN.^22,23^ Interestingly, the population of the Tg^+^ conformer in α-GlcNAc is significantly higher than in α-Glc and α-GlcN. While GlcN and Glc prefer the G^–^g^+^ and G^+^g^–^ gauche configuration, the population of the G^–^g^+^ conformer in α-GlcNAc has dropped by about 20%. It suggests that the gauche effect, typically attributed to the G^–^g^+^ and G^+^g^–^ conformers of Glc and GlcN, is attenuated in the N-acetylated sugar α-GlcNAc.

**Figure 5:**
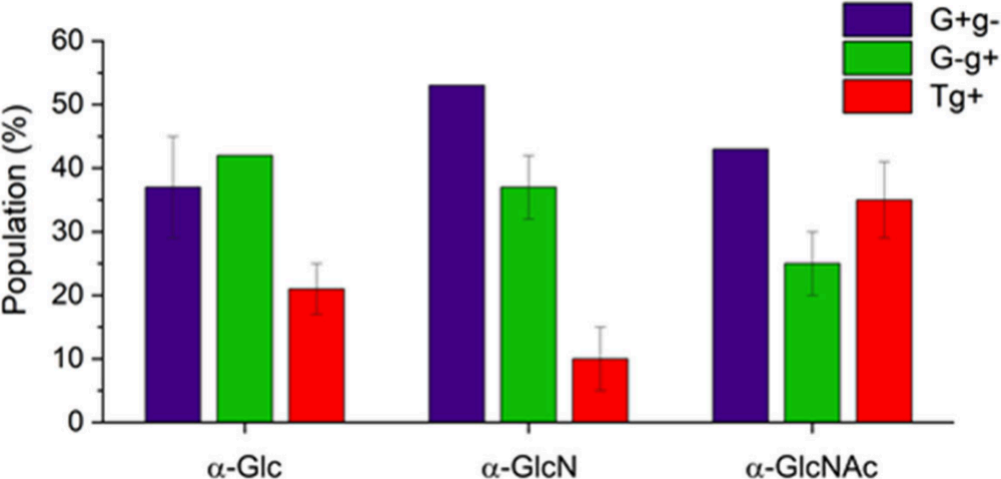
Experimental relative abundance of counterclockwise conformers of α-GlcNAc, α-GlcN, and α-Glc. The error bars represent the standard deviation of the values obtained at different transitions, common to the three conformers. Note that the most populated conformer is used as a reference, so it does not have an associated error.

To account for all the above, a complete set of calculations of the intramolecular H-bond strength was performed using the second-order perturbation theory of Natural Bond Orbital (NBO) analysis.^42^ The results are presented in Table S10 and Figures S4 and S5 of the SI. Based on the calculations summarized in Table 2, the interaction that is significantly different between the three molecules (namely α-Glc, α-GlcN, and α-GlcNAc) is the O_3_H···X interactions (X = O_2_H, NH_2_, or O=C). For α-Glc and α-GlcN, the respective n(O_2_H) → σ*(O_3_H) and n(NH_2_) → σ*(O_3_H) interactions do not have a significant dependence on the hydroxymethyl configuration. However, in α-GlcNAc, the corresponding n(C=O) → σ*(O_3_H) interaction has the highest energy (25 kJ/mol) for the Tg^+^ conformer. As shown above, the hydroxymethyl group in Tg^+^ is involved in the unique three cooperative hydrogen bonding network that stabilizes this species, increasing its population with respect to those of α-Glc and α-GlcN according to the increment of n(C=O) → σ*(O_3_H) interaction energy. Curiously, although acetamido and hydroxymethyl groups are relatively far away and in the opposite position within the α-GlcNAc pyranose ring (R_C2-__C6_ > 4 Å), relative conformational population measurements and a NBO analysis prove that it exists a strong connexion between both groups through a robust intramolecular hydrogen bond network.

**Table 2:** Natural Bond Orbital (NBO) Analysis of Experimental *Counterclockwise* Conformers of α-Glc, α-GlcN, and α-GlcNAc Using B2PLYP-D3BJ/6-311++G(d,p) Theory Level

	Second-Order Perturbation Theory Analysis of Fock Matrix in NBO Basis		
	E(2) (kJ/mol)		
	α-GlcNAc	α-GlcN	α-Glc
Structure	n(C=O)→σ*(O_3_H)	n(NH_2_)→σ*(O_3_H)	n(O_2_H)→σ*(O_3_H)
G^–^g^+^	16.8	7.2	1.6
G^+^g^–^	21.0	7.0	1.6
Tg^+^	25.2	6.7	1.4

Our findings indicate that all the observed conformers have the same orientation of the acetamido group, which is related to the biological role of this moiety. Previous studies on chicken hepatic lectin (CHL) have revealed a nearly complete preference for GlcNAc over monosaccharide ligands.^43^ Interestingly, it does not show any affinity for smaller sugars like d-glucose and 2-deoxyglucose, which cannot be explained using an exclusion mechanism alone. Specific residues, such as tyrosine and valine, form a binding pocket for the acetamido group, which may explain the high selectivity of GlcNAc binding. Therefore, the unique affinity for GlcNAc strongly relies on the selective formation of additional contacts between different amino acid residues and the acetamido group. Moreover, the orientation of the acetamido group is critical in the specific recognition process of bisected GlcNAc-containing N-glycans. For example, in the dendrinic cell receptors, the binding modes and the orientation of the acetamido group change drastically the affinity of the lectins with the glycans.^44^

In summary, we have uncovered the actual molecular shape of α-GlcNAc, an essential chemical scaffold in glycobiology, by employing a cutting-edge combination of high-resolution rotational spectroscopy, pulsed supersonic expansion, and laser ablation techniques, providing three structures that can serve as a basis to represent the conformational behavior of GlcNAc and rationalize its biological behavior. They all present the same orientation of the acetamido group, which aligns perfectly with α-GlcNAc’s highly selective binding under biological conditions. By deciphering the molecular shape of α-GlcNAc in isolation conditions, we have discovered an intramolecular hydrogen bonding network that connects the hydroxymethyl and acetamido groups responsible for the unusual prevalence of the Tg^+^ in the conformational distribution. This discovery challenges the previously accepted solvent-dependent phenomenon known as the gauche effect. α-GlcNAc provides an excellent model system for examining the effects of intramolecular hydrogen bonding on the bare molecule. This study is a step forward in this direction.

Overall, this study represents a significant step forward in understanding the molecular behavior of α-GlcNAc and its role in glycobiology. The new experimental information makes recognizing the conformational space differences between α-GlcNAc and related α-Glc and α-GlcN straightforward. The insights from this research may inspire future studies and highlight the importance of exploring different experimental methods and environments in scientific research.

## Supporting Information Available

Additional experimental details for obaining the microwave spectrum; Tables S1–S3 containing the theoretical results for the most stable conformers at different levels of theory; Figure S1 showing the most stable structures at B3LYP-D3BJ/6-311++G(d,p) level of theory; Figure S2 with the complete and raw microwave spectrum; Tables S4–S6 with the measured transitions for the identified conformers; Tables S7–S9 with the Cartesian coordinates for the identified conformers; Table S10 with NBO analysis of the all intramolecular H-bond; and Figures S4 and S5 with the NCI-plots and reduced electronic density gradient for the observed conformers (PDF)
