# Supplementary material for: Revealing the Structure of Sheer N-Acetylglucosamine, an Essential Chemical Scaffold in Glycobiology
Source: J Phys Chem Lett. 2024 Oct 7;15(41):10314–20. doi: 10.1021/acs.jpclett.4c02128 (PMC11492373; doi:10.1021/acs.jpclett.4c02128)

SUPPLEMENTARY INFORMATION for

REVEALING THE STRUCTURE OF SHEER N-ACETYLGLUCOSAMINE, AN ESSENTIAL CHEMICAL SCAFFOLD IN GLYCOBIOLOGY.

Elena R. Alonso[†], Aran Insausti[#], Isabel Peña[◊], Miguel Sanz-Novo[†], Raúl Aguado[†], Iker León[†], José L. Alonso[†].*

† Grupo de Espectroscopia Molecular (GEM), Edificio Quifima, Área de Química-Física, Laboratorios de Espectroscopia y Bioespectroscopia, Parque Científico UVa, Unidad Asociada CSIC, Universidad de Valladolid, 47011 Valladolid (Spain).

# Departamento de Química Física, Facultad de Ciencia y Tecnología, Universidad del País Vasco, Barrio Sarriena s/n, 48940 Leioa (Spain).

◊ Departamento de Química Física y Química Inorgánica, Facultad de Ciencias, Universidad de Valladolid, 47011 Valladolid (Spain).

Experimental Methods:

N-acetyl-D-glucosamine (m.p. 211ºC) is a thermally fragile molecule that cannot be transferred intact into the vapor phase using conventional heating methods. A laser ablation chirped pulse Fourier transform microwave (LA-CP-FTMW) spectrometer, described in detail elsewhere[1–3] was used to obtain rotational signatures. The experimental procedure involved grinding finely powdered α-GlcNAc and mixing it with a small amount of a commercial binder. The mixture was then pressed into cylindrical rods that were held at the exit of a special pulsed nozzle, where they are ablated using the fourth harmonic (266nm) of a pico-second Nd:YAG laser (~10mJ). The vaporized products were seeded in neon at a stagnation pressure of 10 bar and expanded adiabatically into the vacuum chamber of the spectrometer. High-power chirped microwave excitation pulses (4 μs), in the 6 to 12 GHz range polarize the molecules in this spectral region. The subsequent transient molecular emission was recorded during 10μs in the time domain using a 50 GS/s oscilloscope at a repetition rate of 2 Hz. Up to 115k free induction decays were averaged, and Fourier transformed to obtain the broadband frequency domain spectrum shown in Figures 2a and S2.

(1) Alonso, E. R.; León, I.; Alonso, J. L. The Role of the Intramolecular Interactions in the Structural Behavior of Biomolecules: Insights from Rotational Spectroscopy. In *Intra- and Intermolecular Interactions Between Non-covalently Bonded Species*; Elsevier, 2021; pp 93–141. https://doi.org/10.1016/b978-0-12-817586-6.00004-9.

(2) Shipman, S. T.; Pate, B. H. New Techniques in Microwave Spectroscopy. In *Handbook of High-resolution Spectroscopy*; John Wiley & Sons, Ltd: Chichester, UK, 2011. https://doi.org/10.1002/9780470749593.hrs036.

(3) Brown, G. G.; Dian, B. C.; Douglass, K. O.; Geyer, S. M.; Shipman, S. T.; Pate, B. H. A Broadband Fourier Transform Microwave Spectrometer Based on Chirped Pulse Excitation. *Rev. Sci. Instrum.* **2008**, *79*, 53103.

Table S1. Theoretical results of the most stable conformers using B3LYP-GD3BJ/6-311++G(d,p). The next most stable structure is predicted at $\Delta E^{ZPE} \approx 900 cm^{-1}$. In the labels, we add *c* in the conformers with for with clockwise H-bond direction.

Conf.	A[a]	B	C	μ_a[b]	μ_b	μ_c	χ_{aa}[c]	χ_{bb}	χ_{cc}	ΔE^{ZPE}[d]	ΔG[e]

Tg^+	1197	353	294	3.7	4.0	0.4	2.19	-0.72	-1.47	0	22
G^+g^-	1137	357	294	2.7	4.5	0.2	2.40	-1.14	-1.26	51	0
G^-g^+	1076	367	317	2.4	5.5	1.5	2.45	-1.16	-1.29	110	80
G^-g^-	1125	364	306	2.4	4.0	2.0	2.38	-0.41	-1.97	590	508
G^+t	1155	355	292	0.3	3.5	0.0	2.34	-0.81	-1.54	708	580
G^-g^+ *[f]	1034	355	320	2.8	2.2	4.4	2.43	-4.34	-1.91	794	498
G^+g^+	1156	352	291	1.6	3.5	1.8	2.36	-0.86	-1.49	794	666
G^+g^- *	1063	350	296	3.0	1.7	2.9	2.36	-4.02	1.67	808	505
G^-g^+ / *c*	1140	384	332	1.7	1.9	2.9	2.42	-3.81	1.39	823	832
Tg^+ *	1201	335	293	3.3	1.4	3.0	2.41	-3.77	1.35	839	609
G^-t	1091	366	315	0.0	4.4	1.4	2.41	-0.83	-1.57	886	753
Tg^- / *c*	1176	374	323	1.1	0.9	2.1	2.27	-3.65	1.37	894	982

[a] A, B and C are predicted equilibrium rotational constants in MHz. [b] μ_a, μ_b and μ_c are the absolute values of the electric dipole moment components in Debyes. [c] χ_{aa}, χ_{bb} and χ_{cc} are the ^{14}N nuclear quadrupole coupling constants.[d] Relative zero-point vibrational corrected energies in cm^{-1}. [e] Gibbs energies calculated at 298 K in cm^{-1}. [f] The structures labeled with "*" the O_3H have an H-bond with lone pair electrons of the amide nitrogen.

Table S2. Theoretical results of the most stable conformers using B2PLYP-GD3BJ/6-311++G(d,p) level theory. The next most stable structure is predicted at $\Delta E^{ZPE} \approx 900$cm^{-1}. In the labels, we add *c* in the conformers with a clockwise H-bond direction.

**Conf.**	**A**[a]	**B**	**C**	μ_a[b]	μ_b	μ_c	χ_{aa}[c]	χ_{bb}	χ_{cc}	ΔE^{ZPE}[d]	ΔG[e]
G^+g^-	1141	358	296	2.8	4.4	0.3	2.37	-1.21	-1.15	0	0
Tg^+	1201	354	295	3.6	4.0	0.4	2.16	-0.78	-1.39	9	79
G^-g^+	1078	368	319	2.4	5.5	1.6	2.41	-1.24	-1.17	45	62
G^-g^-	1127	365	308	2.3	4.0	2.0	2.35	-0.47	-1.88	548	533
G^-g^+ *[f]	1037	356	321	2.8	2.2	4.4	2.39	-4.26	1.87	644	406
G^+t	1158	356	293	0.3	3.5	0.0	2.31	-0.87	-1.44	647	566
G^+g^- *	1062	351	297	3.1	1.6	2.8	2.31	-3.97	1.66	664	424
Tg^+ *	1204	336	294	3.4	1.3	2.9	2.38	-3.75	1.37	751	577
G^+g^+	1159	353	293	1.7	3.4	1.9	2.32	-0.93	-1.39	768	690
G^-g^+ / *c*	1143	386	333	1.7	1.9	2.9	2.39	-3.74	1.35	838	919

[a] A, B and C are predicted equilibrium rotational constants in MHz. [b] μ_a, μ_b and μ_c are the absolute values of the electric dipole moment components in Debyes. [c] χ_{aa}, χ_{bb} and χ_{cc} are the ^{14}N nuclear quadrupole coupling constants.[d] Relative zero-point vibrational corrected energies in cm^{-1}. [e] Gibbs energies calculated at 298 K in cm^{-1}. [f] The structures labeled with "*" the O_3H have an H-bond with lone pair electrons of the amide nitrogen.

Table S3. Theoretical results of the experimentally fitted conformers using MP2/6-311++G(d,p) level theory.

**Conf.**	**A**[a]	**B**	**C**	μ_a[b]	μ_b	μ_c	χ_{aa}[c]	χ_{bb}	χ_{cc}	ΔE^{ZPE}[d]	ΔG[e]
Tg^+	1208	355	297	3.6	3.8	0.5	2.09	-0.85	-1.24	91	174
G^+g^-	1146	359	298	2.8	4.3	0.3	2.28	-1.32	-0.97	0	0
G^-g^+	1081	370	322	2.4	5.3	1.6	2.33	-1.38	-0.95	8	21

[a] A, B and C are predicted equilibrium rotational constants in MHz. [b] μ_a, μ_b and μ_c are the absolute values of the electric dipole moment components in Debyes. [c] χ_{aa}, χ_{bb} and χ_{cc} are the ^{14}N nuclear quadrupole coupling constants.[d] Relative zero-point vibrational corrected energies in cm^{-1}. [e] Gibbs energies calculated at 298 K in cm^{-1}.

Figure S1. The most stable structures of α-GlcNAc at B3LYP-GD3BJ/6-311++G(d,p) level of theory. H-bond distances have been added (in Angstrom).

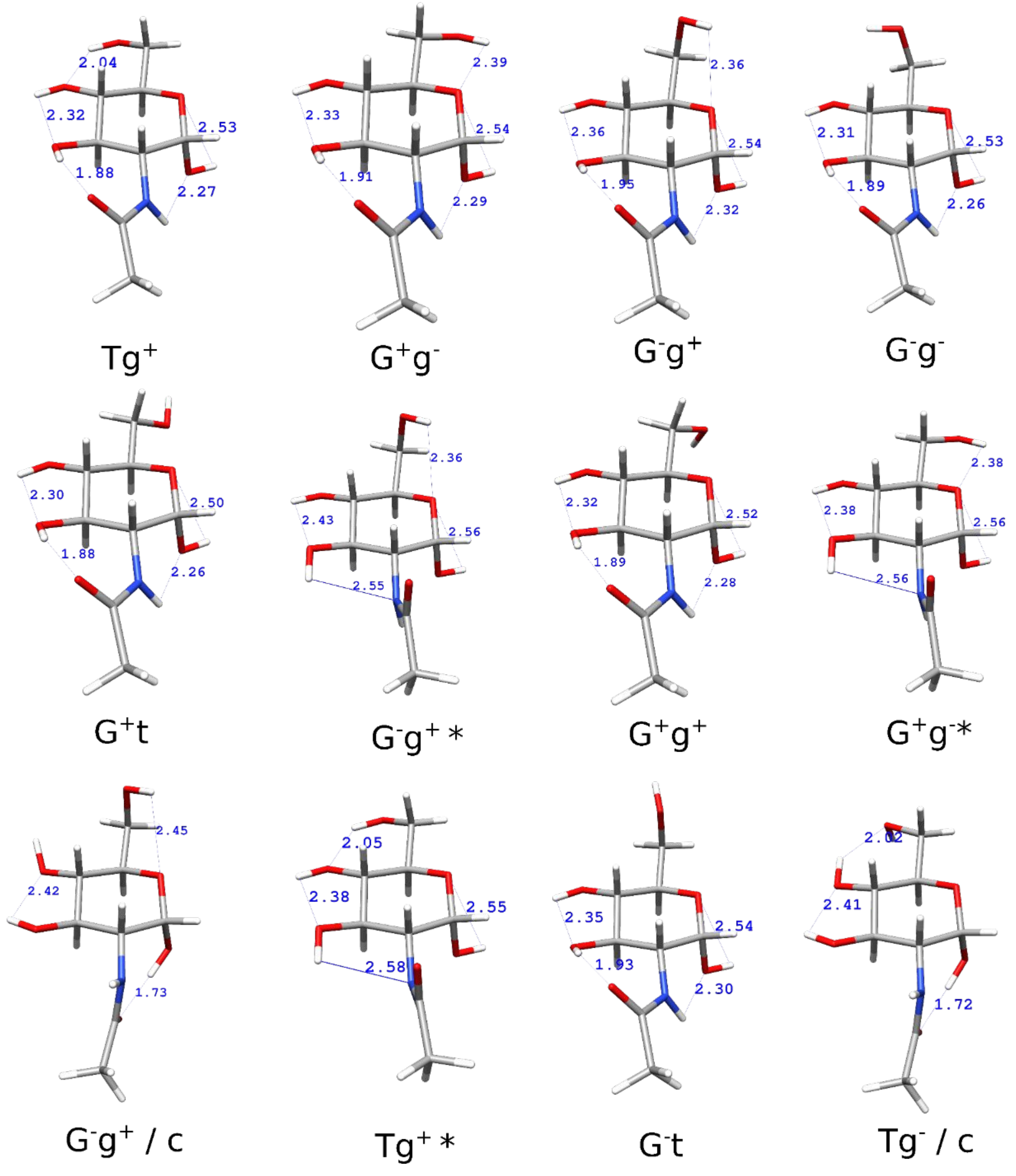

Figure S2. Broadband LA-CP-FTMW Rotational Spectra without removing the fragmentation lines.

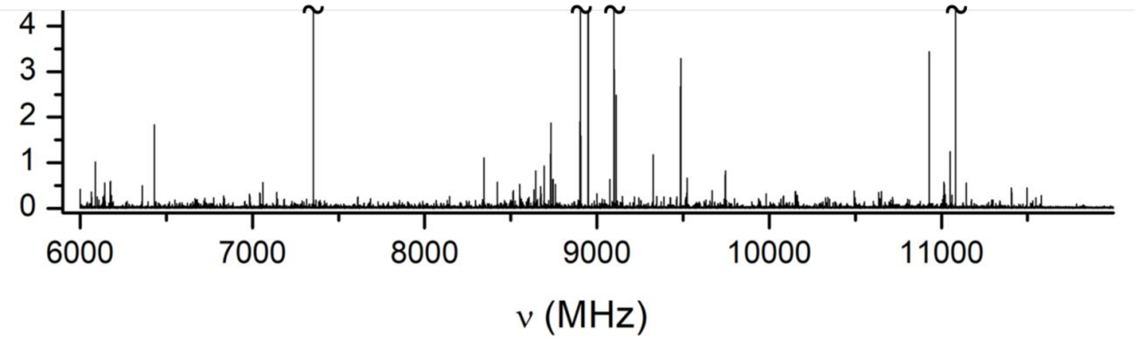

Table S4. Measured frequencies for the rotational transition of conformer G+g- of α-GlcNAc (in MHz)

J'	*K'*$_{-1}$	*K'*$_{+1}$	*J"*	*K"*$_{-1}$	*K"*$_{+1}$	ν_{obs}	$\nu_{obs} - \nu_{cal}$
10	0	10	9	1	9	6041.7103	0.0031
11	0	11	10	1	10	6671.0399	0.0014
12	0	12	11	1	11	7289.4313	0.0071
13	0	13	12	1	12	7899.7222	0.0026
14	0	14	13	1	13	8504.2536	0.0111
15	0	15	14	1	14	9104.7452	-0.0109
16	0	16	15	1	15	9702.5435	-0.0004
18	0	18	17	1	17	10893.2769	0.0003
10	1	10	9	0	9	6272.7677	-0.0241
11	1	11	10	0	10	6828.9725	-0.0037
12	1	12	11	0	11	7395.4484	-0.0038
13	1	13	12	0	12	7969.8535	-0.0145
14	1	14	13	0	13	8550.1090	0.0108
15	1	15	14	0	14	9134.4452	0.0133
16	1	16	15	0	15	9721.5908	0.0050
17	1	17	16	0	16	10310.6501	0.0106
18	1	18	17	0	17	10900.9554	0.0018
7	2	6	6	1	5	6393.6155	0.0148
8	2	7	7	1	6	6844.2809	0.0211
9	2	8	8	1	7	7276.1903	-0.0170
10	2	9	9	1	8	7696.2298	0.0285
13	2	12	12	1	11	8969.6794	0.0221
14	2	13	13	1	12	9425.3340	0.0186
15	2	14	14	1	13	9904.4458	-0.0089
16	2	15	15	1	14	10407.2188	0.0003
17	2	16	16	1	15	10931.3756	-0.0059
14	1	13	13	2	12	8562.6077	0.0064
15	1	14	14	2	13	9272.1885	0.0116
16	1	15	15	2	14	9954.0079	-0.0032
17	1	16	16	2	15	10612.6408	-0.0072
18	1	17	17	2	16	11252.8860	-0.0012
8	3	6	7	2	5	9068.8513	-0.0095
9	3	7	8	2	6	9571.0943	-0.0091
10	3	8	9	2	7	10031.9171	-0.0282
11	3	9	10	2	8	10454.0496	-0.0306
14	3	12	13	2	11	11541.2567	-0.0106
4	4	1	3	3	0	8345.0412	-0.0397
4	4	0	3	3	1	8345.0412	-0.0397
5	4	1	4	3	2	9000.2455	-0.0470
5	4	2	4	3	1	8999.4352	0.0205
6	4	2	5	3	3	9655.5017	0.0202

7	4	3	6	3	4	10311.1920	0.0197
11	0	11	10	0	10	6765.2780	-0.0182
12	0	12	11	0	11	7353.1038	-0.0002
13	0	13	12	0	12	7942.0995	0.0318
14	0	14	13	0	13	8532.0406	-0.0022
15	0	15	14	0	14	9122.8308	0.0194
10	1	10	9	1	9	6135.9666	0.0016
11	1	11	10	1	10	6734.7103	-0.0081
15	1	15	14	1	14	9116.3571	-0.0194
10	2	9	9	2	8	6455.3711	-0.0019
12	2	11	11	2	10	7701.6315	0.0093
13	2	12	12	2	11	8317.7950	0.0269
14	2	13	13	2	12	8929.5740	-0.0098
15	2	14	14	2	13	9537.4428	-0.0293
16	2	15	15	2	14	10141.9182	-0.0049
9	2	7	8	2	6	6116.3592	-0.0016
10	2	8	9	2	7	6811.0694	-0.0022
11	2	9	10	2	8	7498.1493	-0.0056
12	2	10	11	2	9	8175.4957	-0.0341
13	2	11	12	2	10	8841.4516	0.0006
14	2	12	13	2	11	9494.2906	-0.0113
7	4	4	6	3	3	10300.5629	0.0268
9	4	6	8	3	5	11571.2972	0.0276
5	5	0	4	4	1	10635.8149	-0.0179
5	5	1	4	4	0	10635.8149	-0.0179
6	5	1	5	4	2	11290.8314	0.0326
6	5	2	5	4	1	11290.8314	0.0326
8	3	5	7	2	6	9514.2534	-0.0181
9	3	6	8	2	7	10294.5462	0.0127
9	1	8	8	1	7	6035.3830	0.0038
10	1	9	9	1	8	6664.5499	-0.0006
11	1	10	10	1	9	7279.9511	-0.0038
12	1	11	11	1	10	7882.3011	0.0049
15	1	14	14	1	13	9639.1681	0.0086

Table S5. Measured frequencies for the rotational transition of conformer G^-g^+ of α-GlcNAc (in MHz).

J'	K'_{-1}	K'_{+1}	*J"*	$K"_{-1}$	$K"_{+1}$	ν_{obs}	$\nu_{obs} - \nu_{cal}$
10	0	10	9	1	9	6441.1213	0.0161
11	0	11	10	1	10	7116.6156	0.0139
12	0	12	11	1	11	7781.7194	0.0116
13	0	13	12	1	12	8438.7773	0.0006
15	0	15	14	1	14	9736.6058	-0.0233
9	1	9	8	0	8	6109.3054	0.0129
10	1	10	9	0	9	6698.6307	0.0173
11	1	11	10	0	10	7298.6045	0.0164
12	1	12	11	0	11	7908.0188	0.0046
13	1	13	12	0	12	8525.1599	0.0134
14	1	14	13	0	13	9148.1994	-0.0050
15	1	15	14	0	14	9775.6140	-0.0200
12	1	11	11	2	10	7430.4414	-0.0006
13	1	12	12	2	11	8238.7461	0.0038
15	1	14	14	2	13	9781.6637	-0.0233
16	1	15	15	2	14	10515.7993	-0.0112
13	2	12	12	1	11	9499.7652	0.0155
14	2	13	13	1	12	10005.6758	-0.0155
15	2	14	14	1	13	10528.9994	-0.0155
4	3	1	3	2	2	6466.8266	0.0238

5	3	2	4	2	3	7164.8942	-0.0063
8	3	5	7	2	6	9342.0409	0.0233
9	3	6	8	2	7	10117.7601	0.0092
4	3	2	3	2	1	6453.9357	0.0009
5	3	3	4	2	2	7126.0380	-0.0076
6	3	4	5	2	3	7782.2240	0.0026
5	4	2	4	3	1	8632.5895	0.0031
6	4	3	5	3	2	9318.4809	0.0035
7	4	4	6	3	3	10001.6978	-0.0225
8	4	5	7	3	4	10680.1510	0.0062
9	4	6	8	3	5	11350.5842	-0.0022
5	4	1	4	3	2	8633.1195	-0.0256
6	4	2	5	3	3	9320.7448	0.0201
7	4	3	6	3	4	10008.4785	-0.0120
8	4	4	7	3	5	10697.1045	-0.0036
9	4	5	8	3	6	11387.8722	-0.0047
14	0	14	13	0	13	9124.7683	-0.0081
10	1	9	9	1	8	6987.6967	0.0050
11	1	10	10	1	9	7649.9320	0.0216
14	1	13	13	2	12	9022.9596	-0.0081
14	1	14	13	1	13	9113.2885	-0.0037
9	2	8	8	2	7	6137.6578	0.0257
10	2	9	9	2	8	6807.1943	-0.0018
11	2	9	10	2	8	7798.1037	0.0010

Table S6. Measured frequencies for the rotational transition of conformer Tg+ of α-GlcNAc (in MHz).

J'	*K'*$_{-1}$	*K'*$_{+1}$	*J"*	*K"*$_{-1}$	*K"*$_{+1}$	ν_{obs}	$\nu_{obs} - \nu_{cal}$
10	0	10	9	0	9	6182.4885	-0.0043
11	0	11	10	0	10	6769.1950	0.0061
10	1	10	9	1	9	6130.3364	0.0089
11	1	11	10	1	10	6729.9725	0.0097
12	1	12	11	1	11	7327.8363	-0.0064
13	1	13	12	1	12	7924.3214	0.0084
14	1	14	13	1	13	8519.6656	-0.0217
15	1	15	14	1	14	9114.2231	-0.0111
16	1	16	15	1	15	9708.1578	-0.0148
10	1	10	9	0	9	6314.4087	-0.0255
11	1	11	10	0	10	6861.9237	0.0197
12	1	12	11	0	11	7420.5691	0.0111
15	1	15	14	0	14	9143.7381	-0.0192
16	1	16	15	0	15	9727.9239	0.0109
11	0	11	10	1	10	6637.2409	-0.0065
12	0	12	11	1	11	7263.7407	0.0075
14	0	14	13	1	13	8490.1434	-0.0209
15	0	15	14	1	14	9094.5086	0.0147
9	2	7	8	2	6	6054.2622	0.0025
10	2	8	9	2	7	6745.5931	-0.0067
11	2	9	10	2	8	7431.7925	0.0141
12	2	10	11	2	9	8110.7286	-0.0043
14	2	12	13	2	11	9440.7307	-0.0236
9	1	8	8	1	7	6008.2857	0.0201
10	1	9	9	1	8	6642.8112	0.0192
13	1	12	12	1	11	8476.0612	0.0229
14	1	13	13	1	12	9066.7193	0.0078
11	2	10	10	2	9	7057.4471	0.0051
13	2	12	12	2	11	8297.7909	0.0065

14	2	13	13	2	12	8911.8923	-0.0028
13	3	10	12	3	9	8681.6241	-0.0004
10	3	8	9	3	7	6534.4505	0.0024
11	3	9	10	3	8	7187.7348	-0.0269
12	3	10	11	3	9	7838.9909	-0.0049
13	3	11	12	3	10	8487.4649	-0.0287
15	3	13	14	3	12	9774.0059	0.0173
3	3	0	2	2	1	6352.6783	-0.0175
4	3	1	3	2	2	7008.6429	-0.0103
3	3	1	2	2	0	6349.8172	0.0191
4	3	2	3	2	1	6994.0395	0.0020
6	3	4	5	2	3	8242.5536	0.0035

Table S7. Cartesian coordinates of α-GlcNAc G-g+. The geometry has been optimized at the B2PLYP-GD3BJ/6-311++G(d,p) level of theory.

**α-GlcNAc G-g+**			
	Coordinate (Angstroms)		
Atom	X	Y	Z
C	-2.09879	0.29678	-0.38696
C	-1.32165	-0.99507	-0.17229
C	0.13760	-0.80348	-0.56762
C	0.72858	0.34690	0.24713
C	-0.12815	1.60651	0.09565
H	-2.09164	0.54564	-1.45375
H	-1.37399	-1.27241	0.88804
H	0.17758	-0.55388	-1.63349
H	0.71962	0.06709	1.30412
H	0.17609	2.35986	0.82678
O	-1.48255	1.36518	0.36842
C	-3.53497	0.22914	0.10441
H	-4.04738	1.15860	-0.17110
H	-4.03396	-0.60841	-0.38112
O	-3.61218	0.00385	1.50377
H	-3.12560	0.71832	1.92744
O	0.07990	2.08541	-1.22269
H	-0.39005	2.92094	-1.31293
O	0.83292	-2.03286	-0.39287
H	1.48731	-1.93554	0.31659
O	-1.92751	-1.99569	-0.97562
H	-1.33909	-2.75931	-0.94425
H	2.24874	1.32788	-0.85103
N	2.10372	0.66360	-0.10567
C	3.14953	-0.04794	0.39666
O	2.98040	-0.99028	1.17096
C	4.52769	0.39140	-0.03859
H	5.02208	-0.44900	-0.52747
H	4.51221	1.24718	-0.71425
H	5.10578	0.64516	0.85099

Table S8: Cartesian coordinates of α-GlcNAc G+g-. The geometry has been optimized at the B2PLYP-GD3BJ/6-311++G(d,p) level of theory.

**α-GlcNAc G+g-**			
	Coordinate (Angstroms)		
Atom	X	Y	Z
C	-2.06845	-0.01464	-0.11893
C	-1.16756	-1.22342	0.08029
C	0.23866	-0.90802	-0.41787
C	0.77375	0.31927	0.32243
C	-0.21899	1.48389	0.23533
H	-2.11926	0.24075	-1.18165
H	-1.11291	-1.45597	1.15394
H	0.18302	-0.68961	-1.49030
H	0.89005	0.06412	1.37941
H	0.06145	2.26996	0.94123
O	-1.51422	1.10287	0.61470
C	-3.47117	-0.20853	0.42256
H	-3.94903	-1.02786	-0.11156
H	-3.41326	-0.46804	1.48708
O	-4.27294	0.94708	0.22252
H	-3.82340	1.67404	0.66462
O	-0.16172	1.96932	-1.09522
H	-0.74177	2.73532	-1.15979
O	1.04657	-2.06634	-0.24710
H	1.79364	-1.85841	0.33744
O	-1.72026	-2.31648	-0.63337
H	-1.04204	-3.00278	-0.64390
H	2.07124	1.45732	-0.90250
N	2.06960	0.77031	-0.16356
C	3.22900	0.17978	0.23195
O	3.23714	-0.78109	1.00300
C	4.50472	0.77533	-0.31476
H	5.05941	-0.00660	-0.83460
H	4.33198	1.61177	-0.99265
H	5.11431	1.11301	0.52467

Table S9: Cartesian coordinates of α-GlcNAc Tg+. The geometry has been optimized at the B2PLYP-GD3BJ/6-311++G(d,p) level of theory.

**α-GlcNAc Tg+**			
	Coordinate (Angstroms)		
Atom	X	Y	Z
C	-2.02738	-0.44967	-0.00705
C	-1.30283	0.87834	-0.17513
C	0.11522	0.78386	0.36780

C	0.84754	-0.34241	-0.36536
C	0.02612	-1.63898	-0.34567
H	-2.06160	-0.72393	1.05198
H	-1.25093	1.11233	-1.24779
H	0.06740	0.55120	1.43795
H	0.97308	-0.04729	-1.41101
H	0.43852	-2.35231	-1.06372
O	-1.30101	-1.44683	-0.75278
C	-3.44861	-0.43503	-0.55921
H	-3.44000	-0.01800	-1.57470
H	-3.81294	-1.46135	-0.61659
O	-4.33430	0.27340	0.28925
H	-3.94094	1.14058	0.44873
O	0.11773	-2.15621	0.97248
H	-0.34561	-3.00027	0.99191
O	0.72865	2.05714	0.20810
H	1.56448	1.95684	-0.27806
O	-2.03661	1.89182	0.50448
H	-1.48298	2.68236	0.50295
H	2.23541	-1.34904	0.87420
N	2.17052	-0.62467	0.17480
C	3.25350	0.13720	-0.13108
O	3.16414	1.13343	-0.85122
C	4.57426	-0.31198	0.44740
H	5.00433	0.51242	1.01703
H	4.48619	-1.19082	1.08680
H	5.25254	-0.53512	-0.37762

Table S10: NBO analysis of the all intramolecular H-bond of α-Glc, α-GlcN and α-GlcNAc

	**Structure**	**Second-Order Perturbation Theory Analysis of Fock Matrix in NBO Basis [E(2) (kcal/mol)]**				
		**σ*(O(1)H)···n(O(5))**	**σ*(O(2)H) or σ*(NH)···n(O(1))**	**σ*(O(3)H)···n(O=C) or n(O(2)) or n(N(2))**	**σ*(O(4)H)···n(O(3))**	**σ*(O(6)H)···n(O(5)) or n(O(4))**
**α-Glc**	α-Glc G-g+	0.02	0.28	0.02	0.12	0.2
		n.d.*	0.64	0.39	0.12	0.28
	α-Glc G+g+	0.02	0.29	0.02	0.14	0.37
		n.d.	0.64	0.37	0.17	0.15
	α-Glc Tg+	0.02	0.32	0.02	0.15	2.17
		n.d.	0.68	0.34	0.16	0.86
**α-GlcN**	α-GlcN G-g+	0.03	0.12	1.72	0.08	0.31
		n.d.	0.22	n.d.	0.12	0.23
	α-GlcN G+g-	0.04	0.13	1.67	0.11	0.4
		n.d.	0.21	n.d.	0.15	0.16
	α-GlcN Tg+	0.03	0.13	1.59	0.17	2.29
		n.d.	0.22	n.d.	0.09	0.88
**α-GlcNAc**	α-GlcNAc G-g+	0.03	0.24	1.42	0.18	0.28
		n.d.	0.45	4.01	0.3	0.19
	α-GlcNAc G+g-	0.04	0.27	1.83	0.26	0.34
		n.d.	0.51	5.04	0.25	0.13
	α-GlcNAc Tg+	0.04	0.32	2.22	0.3	2.32
		n.d.	0.61	6.01	0.19	0.75

Footnote:*n.d.: not determined.

Figure S4. NCI plot of the detected conformers of ⍰-GlcNAc at B2PLYP-GD3BJ/6-311++G(d,p) level of theory

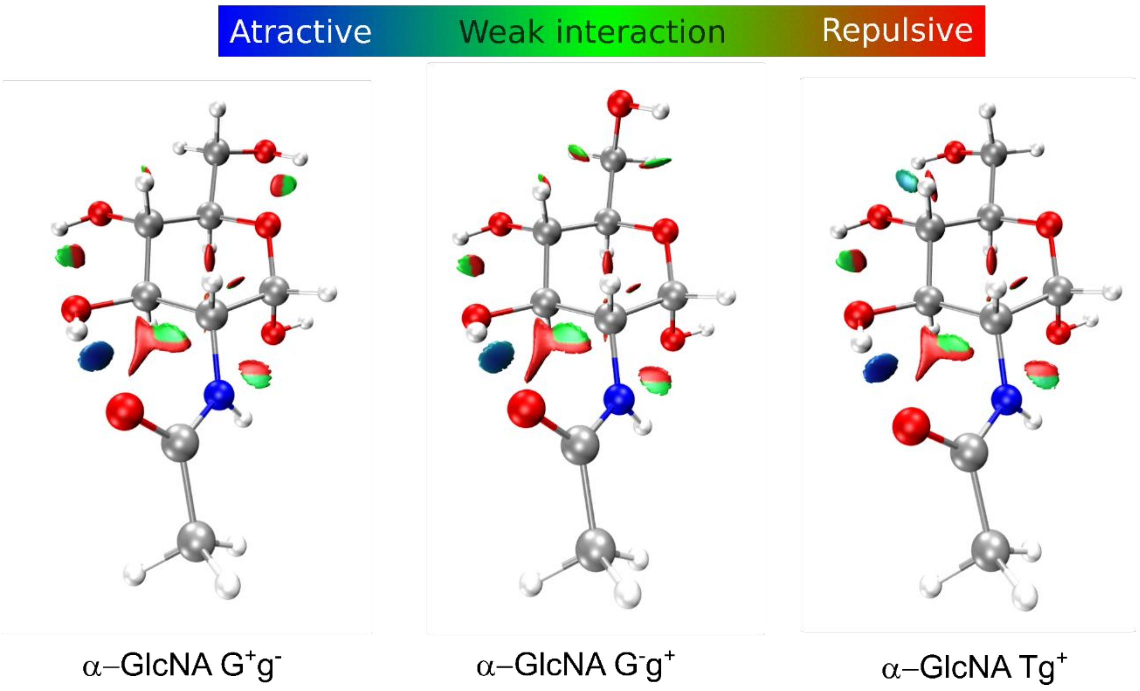

Figure S5. Reduced electronic density gradient for the observed *counterclockwise* G+g- conformers of α-GlcNAc (Red), α-Glc (Green), and α-GlcN (Blue) (all calculations using B2PLYP-GD3BJ/6-311++G(d,p) method). Negative values of sign(λ_2) ρ (a.u.) show attractive non-covalent interactions, while positive regions show nonbonding steric clashes. Values close to zero indicate weak non-covalent interactions. The most attractive non-covalent interaction of each molecule is indicated with the arrow.

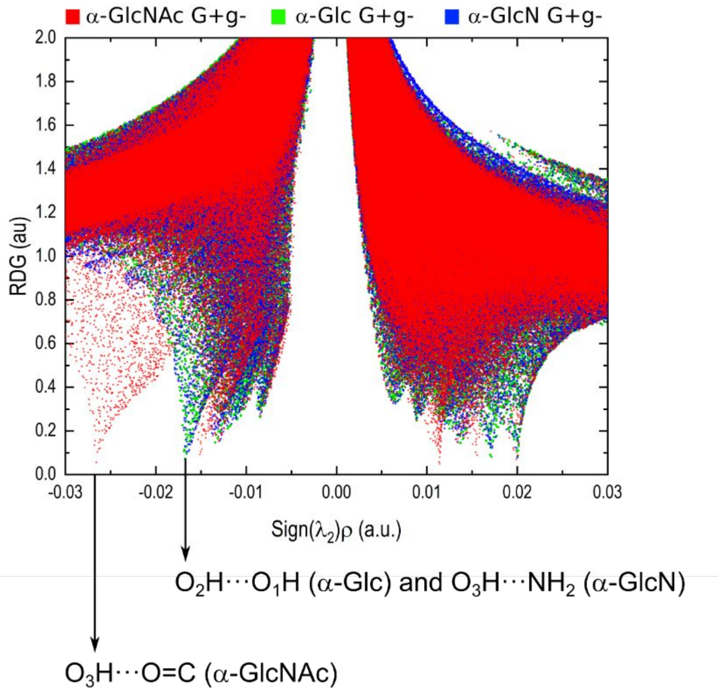

Supplement: Supplementary file 1 — jz4c02128_si_001.pdf [file jz4c02128_si_001.pdf]
